# Comparison of lesion characteristics between conventional and high-power short-duration ablation using contact force-sensing catheter in patients with paroxysmal atrial fibrillation

**DOI:** 10.1186/s12872-021-02196-y

**Published:** 2021-08-09

**Authors:** Chun-Chao Chen, Po-Tseng Lee, Vu Van Ba, Chieh-Mao Chuang, Yenn-Jiang Lin, Li-Wei Lo, Yu-Feng Hu, Fa-Po Chung, Chin-Yu Lin, Ting-Yung Chang, Jennifer Jeanne Vicera, Ting-Chun Huang, Chih-Min Liu, Cheng-I Wu, Isaiah C. Lugtu, Ankit Jain, Shih-Lin Chang, Shih-Ann Chen

**Affiliations:** 1 Division of Cardiology, Department of Medicine, Heart Rhythm Center, Taipei Veterans General Hospital, 201 Sec. 2, Shih-Pai Road, Taipei, Taiwan; 2 Division of Cardiology, Department of Internal Medicine, Shuang Ho Hospital, Taipei Medical University, New Taipei City, Taiwan; 3 Taipei Heart Institute, Taipei Medical University, Taipei, Taiwan; 4 Division of Cardiology, Department of Internal Medicine, School of Medicine, College of Medicine, Taipei Medical University, Taipei, Taiwan; 5 Division of Cardiovascular Medicine, Department of Internal Medicine, National Cheng Kung University Hospital, Tainan, Taiwan; 6 Cardiovascular Centre, E Hospital, Hanoi, Vietnam; 7 Division of Pediatric Cardiology, China Medical University Children’s Hospital, China Medical University, Taichung, Taiwan; 8 Faculty of Medicine, School of Medicine, National Yang Ming Chiao Tung University, Taipei, Taiwan; 9 Department of Medicine, Section of Cardiology, University of Santo Tomas Hospital, Manila, Philippines; 10 Heart Institute, Chinese General Hospital and Medical Center, Manila, Philippines; 11 Vardhman Mahavir Medical College and Safdarjung Hospital, New Delhi, India; 12 Cardiovascular Center, Taichung Veterans General Hospital, Taichung, Taiwan

**Keywords:** Atrial fibrillation, Pulmonary vein isolation, Contact force-guided ablation, Voltage, Force time integral, High-power short-duration ablation

## Abstract

**Background:**

Transmural lesion creation is essential for effective atrial fibrillation (AF) ablation. Lesion characteristics between conventional energy and high-power short-duration (HPSD) setting in contact force-guided (CF) ablation for AF remained unclear.

**Methods:**

Eighty consecutive AF patients who received CF with conventional energy setting (power control: 25–30 W, force–time integral = 400 g s, n = 40) or with HPSD (power control: 40–50 W, 10 s, n = 40) ablation were analyzed. Of them, 15 patients in each conventional and HPSD group were matched by age and gender respectively for ablation lesions analysis. Type A and B lesions were defined as a lesion with and without significant voltage reduction after ablation, respectively. The anatomical distribution of these lesions and ablation outcomes among the 2 groups were analyzed.

**Results:**

1615 and 1724 ablation lesions were analyzed in the conventional and HPSD groups, respectively. HPSD group had a higher proportion of type A lesion compared to conventional group (*P* < 0.01). In the conventional group, most type A lesions were at the right pulmonary vein (RPV) posterior wall (50.2%) whereas in the HPSD group, most type A lesions were at the RPV anterior wall (44.0%) (*P* = 0.04). The procedure time and ablation time were significantly shorter in the HPSD group than that in the conventional group (91.0 ± 12.1 vs. 124 ± 14.2 min, *P* = 0.03; 30.7 ± 19.2 vs. 57.8 ± 21 min, *P* = 0.02, respectively). At a mean follow-up period of 11 ± 1.4 months, there were 13 and 7 patients with recurrence in conventional and HPSD group respectively (*P* = 0.03).

**Conclusion:**

Optimal ablation lesion characteristics and distribution after conventional and HPSD ablation differed significantly. HPSD ablation had shorter ablation time and lower recurrence rate than did conventional ablation.

## Introduction

Complete and durable pulmonary vein (PV) isolation (PVI) with point-by-point adjacent transmural lesion creation is essential in effective atrial fibrillation (AF) ablation [1]. Nontransmural ablation lesions, which are responsible for reconnecting gaps on PVI lines, are associated with clinical recurrence [2]. Contact force-guided (CF) ablation for PVI provides improved clinical outcomes [3, 4]: a force time integral (FTI) of > 392 g s potentially predicts effective creation of transmural lesion [5]. High-power short-duration (HPSD) ablation, reported to be an effective PVI method [6, 7], has a relatively short procedure time and fluoroscopy time [8–10].

Local bipolar electrogram (EGM) voltage is related to the thickness of the atrial musculature. An increase in this voltage indicates conduction gap after linear ablation in the left atrium [11], whereas a significant decrease in this voltage indicates transmural lesion creation during AF ablation [12].

Under the guidance of the ablation index, the acute reconnection rate can be significantly reduced to less than that with conventional ablation [13]. However, the lesion characteristics in conventional energy and HPSD setting in CF ablation for PVI remain unclear. This study aimed to compare lesion characteristics and outcomes between these 2 energy settings using CF ablation in PVI.

## Methods

### Patient population

In total, 80 consecutive patients with paroxysmal AF (55 men; mean age, 59.3 ± 11.0 years) using CF ablation for PVI were included. Of them, 40 patients (mean age, 56.9 ± 10.6 years; 65.0% men) received conventional energy (conventional group) and 40 (mean age, 56.70 ± 11.5 years; 72.5% men) received HPSD ablation (HPSD group). The cardiac structure and function as well as the mean left atrial diameter were assessed through echocardiography. After matching with age and gender, total 15 and 15 patients with initial rhythm as sinus rhythm in conventional and HPSD group were analyzed respectively for the characteristics of ablation lesion after PVI. All patients were informed and their written consents were all obtained. The study was approved by the Joint Institutional Review Board of Taipei Veterans General Hospital (T-TPEVGH No. 50831). All method were carried out in accordance with relevant guidelines and regulations.

### Electrophysiological procedure

We have detailed the mapping and ablation procedures previously [14]. In brief, before the procedure, all patients underwent transesophageal echocardiography and cardiac computed tomography to rule out intracavitary thrombi. The left atrial electroanatomical map was created using Ensite Precision (version 2.0.1; St. Jude Medical, St. Paul, MN, USA, now Abbott). An Inquiry AFocus II (electrode spacing: 3.5–3.5–3.5) spiral catheter or Advisor HD Grid mapping catheter (St. Jude Medical, St. Paul, MN, USA, now Abbott) was used to record PV potentials (PVPs). PVI was performed in the antrum circumferential ablation pattern by using a TactiCath Quartz Contact Force Ablation Catheter (St. Jude Medical, St. Paul, MN, USA, now Abbott). Voltage map was created before and after pulmonary vein ablation. The inter-lesion distance in both conventional and HPSD group was ≤ 5 mm. Both conventional and HPSD group were performed with non-steerable long sheath (Swartz™ Braided Transseptal Guiding Introducers, St. Jude Medical, St. Paul, MN, USA, now Abbott).

In the conventional group, the power setting was 30 W, with limited ranges of 25 W for the posterior wall near the esophagus. The irrigation flow rate during RF application was 17 to 30 mL/min. Point-by-point ablation was performed. For each point, the FTI goal was 400 g s. In the HPSD group, the power setting was 50 W for the anterior walls and 40 W for the posterior walls. Each point ablation time was set as 10 s. When performing HPSD ablation, the contact force was at least 10 g in each point. During ablation, bipolar EGM voltage was observed and recorded. Additional ablation energy was applied if bipolar EGM voltage did not meet the transmural criteria.

The PVI endpoint was PVP elimination. The interference of far-field signals was excluded using superior vena cava and left atrial appendage pacing maneuver. PV reconnection was re-checked after a waiting period of 30 min. Gaps were defined as the areas of acute PV reconnection. If PVP recurred, additional ablation was applied at the gap to eliminate the residual PVP.

### Definition of ablation lesion type according to EGM voltage reduction and morphology

**Fig. 1:**
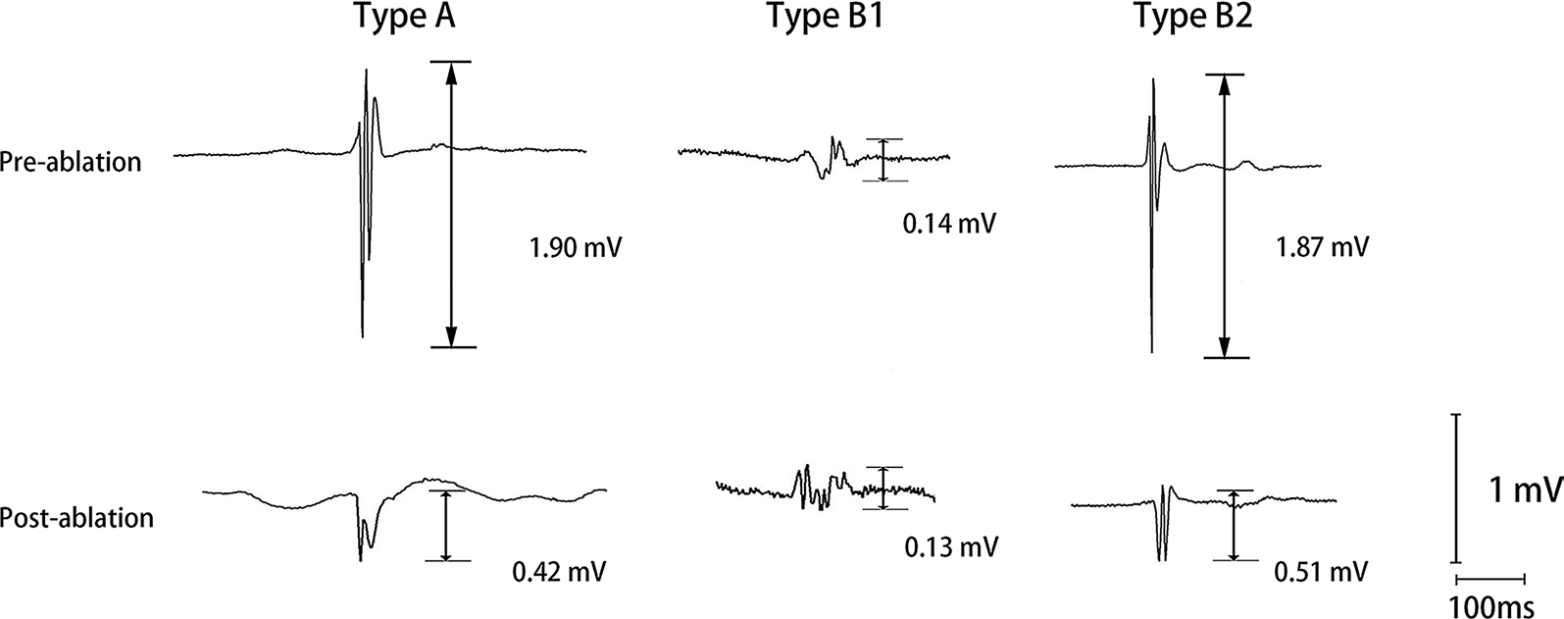
Examples of type A and type B lesions

The amplitude and morphology of bipolar EGM voltage (pre- and post-ablation) and FTI were recorded at each point in the anterior and posterior wall of the left superior PV (LSPV), left inferior PV (LIPV), right superior PV (RSPV), and right inferior PV (RIPV) during ablation. An optimal ablation (i.e., type A) lesion was defined as (a) QR morphology with disappearance of positivity after ablation, (b) QRS morphology with diminution of > 75% of positivity, or (c) RSR′ morphology with the disappearance of R′ positivity [12] (Fig. 1). Moreover, type B ablation lesions, defined as having ablation points other than type A lesions, could be divided into 2 subgroups: (a) with ablation points located at the low bipolar EGM voltage area too small to analyze (Fig. 1) and (b) lesions with obvious QR, QRS, and RSR′ morphology not meeting the transmural criteria after ablation (Fig. 1).

The aforementioned bipolar EGM criteria were validated by an electrophysiologist blinded to the procedures.

### Follow-up of AF recurrence events

The situations of patients were tracked 2 weeks after the catheter ablation and then every 3 months thereafter at our cardiology clinic or by the referring physicians. Antiarrhythmic drugs were prescribed during the blanking period to prevent any recurrence of AF (blanking period was defined as < 3 months after the ablation). When the patients were experiencing symptoms suggestive of a tachycardia after the ablation, a 24-h Holter monitoring or a cardiac event recording would be performed to define the cause of the clinical symptoms. Regular 24-h Holter monitoring or cardiac event recording was performed every 3 months. If more than one episode of recurrent symptomatic AF was documented after the blanking period, the patients were encouraged to receive a second ablation procedure, or antiarrhythmic drugs would be prescribed to control such recurrent AF. AF recurrence was defined as an episode, confirmed by an electrocardiogram, lasting for > 30 s after ablation.

### Statistical analysis

Here, continuous variables are expressed as means and standard deviations. Student’s *t* test or Mann–Whitney *U* test was used for unpaired group comparison, whereas the Chi-square test was used for evaluating associations between categorical variables; when the sample size was too small for chi-squared test results to be valid, Fisher’s exact test was used. A *P* of < 0.05 was considered to indicate statistical significance. All statistical analysis was performed on SPSS (version 23; IBM, Armonk, NY, USA).

## Results

### Baseline characteristics of study population

There was no significant difference in baseline characteristics between the 2 study groups (Table 1).

**Table 1:** Baseline characteristics of patients with atrial fibrillation

	HPSD (n = 40)	Conventional (n = 40)	*P* value
Age	56.9 ± 10.6	56.70 ± 11.5	0.56
Sex (male, %)	26 (65.0)	29 (72.5)	0.59
Hypertension	10 (25.0)	8 (20.0)	0.56
Diabetes mellitus	2 (5.0)	3 (7.5)	0.64
Stroke	0 (0)	0 (0)	1.00
CHA2DS2-VASc	2.1 ± 1.10	2.2 ± 1.00	0.59
LA diameter (mm)	36.4 ± 4.30	35.4 ± 4.63	0.71
LV ejection fraction (%)	64.2 ± 6.9	66.4 ± 5.3	0.67
Atrial fibrillation type			
Paroxysmal	30 (75.0)	25 (62.5)	0.12
Persistent	10 (25)	15 (37.5)	0.43

*HPSD* high-power short-duration, *CF* contact force, *LA* left atrium, *LV* left ventricle

### Ablation lesion characteristics

**Fig. 2:**
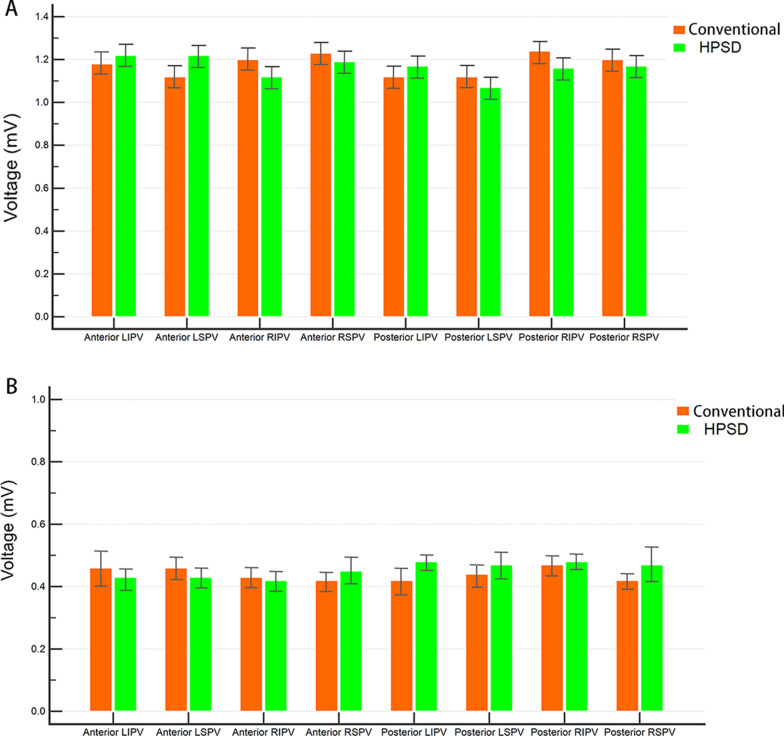
Mean bipolar EGM voltage of different pulmonary vein (PV) region (**A**) before and (**B**) after ablation. HPSD = high-power short-duration; LIPV = left inferior PV; LSPV = left superior PV; RIPV = right inferior PV; RSPV = right superior PV

In total, 1615 and 1724 ablation points were enrolled in the conventional and HPSD groups, respectively. However, the mean bipolar EGM voltage of different PV regions did not differ significantly between the 2 groups (Fig. 2A); moreover, the post-ablation voltage reduction remained similar between these groups (Fig. 2B).

**Fig. 3:**
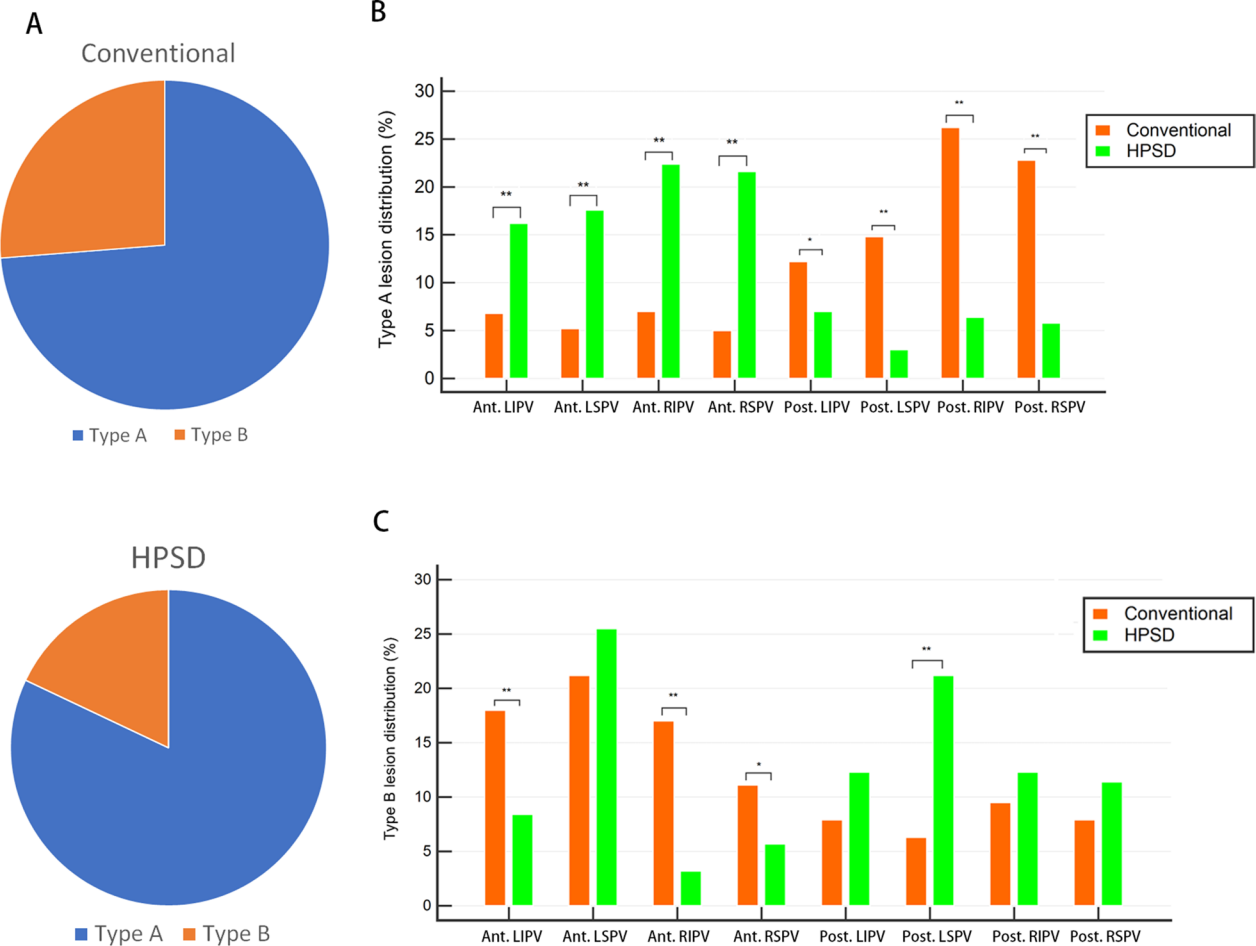
**A** Proportion of type A lesion. **B** Distribution of type A lesions. **C** Distribution of type B lesions. *P < 0.05, **P < 0.01

The conventional and HPSD groups respectively presented 144 (10.5%) and 206 (11.9%) lesions with very low bipolar voltage before ablation (< 0.2 mV), which were difficult to measure the reduction of voltage post-ablation. Therefore, those lesions were excluded for analysis. The proportion of ablation lesion types among the groups is illustrated in Fig. 3A: the proportion of type A lesions was significantly higher in the HPSD group than in the conventional group (82.0% vs. 72.1%, *P* < 0.01). In the conventional group, 1121 (91.6%) and 102 (8.3%) lesions had an FTI of ≥ 400 and < 400 g s, respectively. Of the lesions with FTI ≥ 400 g s, 784 (69.9%) were type A lesions.

The distribution of type A lesion significantly differed between the 2 groups (*P* = 0.04) (Fig. 3B): anterior walls of LSPV (conventional vs. HPSD: 5.2% vs. 17.6%, *P* < 0.01), LIPV (conventional vs. HPSD: 6.8% vs. 16.2%, *P* < 0.01), RSPV (conventional vs. HPSD: 5% vs. 21.6%, *P* < 0.01), and RIPV (conventional vs. HPSD: 7% vs. 22.4%, *P* < 0.01) and posterior walls of LSPV (conventional vs. HPSD: 14.8% vs. 3.0%, *P* < 0.01), LIPV (conventional vs. HPSD: 12.2% vs. 7.0%, *P* = 0.04), RSPV (conventional vs. HPSD: 22.8% vs. 5.8%, *P* < 0.01), and RIPV (conventional vs. HPSD: 26.2% vs. 6.4%, *P* < 0.01). Similarly, type B lesion distribution significantly differed between the 2 groups (*P* = 0.04) (Fig. 3C): anterior walls of LSPV (conventional vs. HPSD: 21.2% vs. 25.5%, *P* = 0.24), LIPV (conventional vs. HPSD: 18.0% vs. 8.4%, *P* < 0.01), RSPV (conventional vs. HPSD: 11.1% vs. 5.7%, *P* = 0.01), and RIPV (conventional vs. HPSD: 17.0% vs. 3.2%, *P* < 0.01) and posterior walls of LSPV (conventional vs. HPSD: 6.3% vs. 21.2%, *P* < 0.01), LIPV (conventional vs. HPSD: 7.9% vs. 12.3%, *P* = 0.10), RSPV (conventional vs. HPSD: 7.9% vs. 11.4%, *P* = 0.18), and RIPV (conventional vs. HPSD: 9.5% vs. 12.3%, *P* = 0.31).

### Gap characteristics

In the conventional and HPSD groups, 79 and 21 gaps were identified 30 min after ablation in 9 (60%) and 6 (40%) patients, respectively (*P* = 0.28). The distribution of gap was not significantly differed in conventional and HPSD group: anterior wall of LSPV (30% vs. 9.5%, *P* = 0.06), posterior wall of LSPV (8.8% vs. 23.8%, *P* = 0.06), anterior wall of LIPV (10.1% vs. 9.5%, *P* = 0.93), posterior wall of LIPV (7.5% vs. 19.0%, *P* = 0.11), anterior wall of RSPV (15.1% vs. 4.7%, *P* = 0.20), posterior wall of RSPV (7.5% vs. 19.0%, *P* = 0.11), anterior wall of RIPV (12.6% vs. 4.7%, *P* = 0.30), posterior wall of RIPV (7.5% vs. 9.5%, *P* = 76). The proportion of gaps lesions was higher in type B lesions than in type A lesions in conventional and HPSD groups (82.3% vs 17.7%, *P* = 0.02. and 80.9% vs 19.1%, *P* = 0.02). The average number of additional ablations in each gap to achieve pulmonary vein isolation were similar between the conventional group and HPSD group (1.94 ± 0.64 vs. 1.80 ± 0.43 additional ablation per gap, *P* = 0.43).

### FTI characteristics

For the type A lesions, the mean FTI was similar between the area with and without gaps (315 ± 58 vs. 295 ± 64 g s, *P* = 0.09). The mean FTI in type A lesions fitting the bipolar EGM voltage criteria was 241 ± 86 g s. However, in the type B lesions, lower FTI was associated with gap formation (311 ± 69 and 287 ± 49 g s for with gap formation and no gap formation, respectively; *P* = 0.03). Moreover, the proportion of gap formation was higher in lesions with FTI < 400 g s than in those with FTI ≥ 400 g s (24.5% vs. 7.0%, *P* < 0.01).

### Procedure results and outcome

**Fig. 4:**
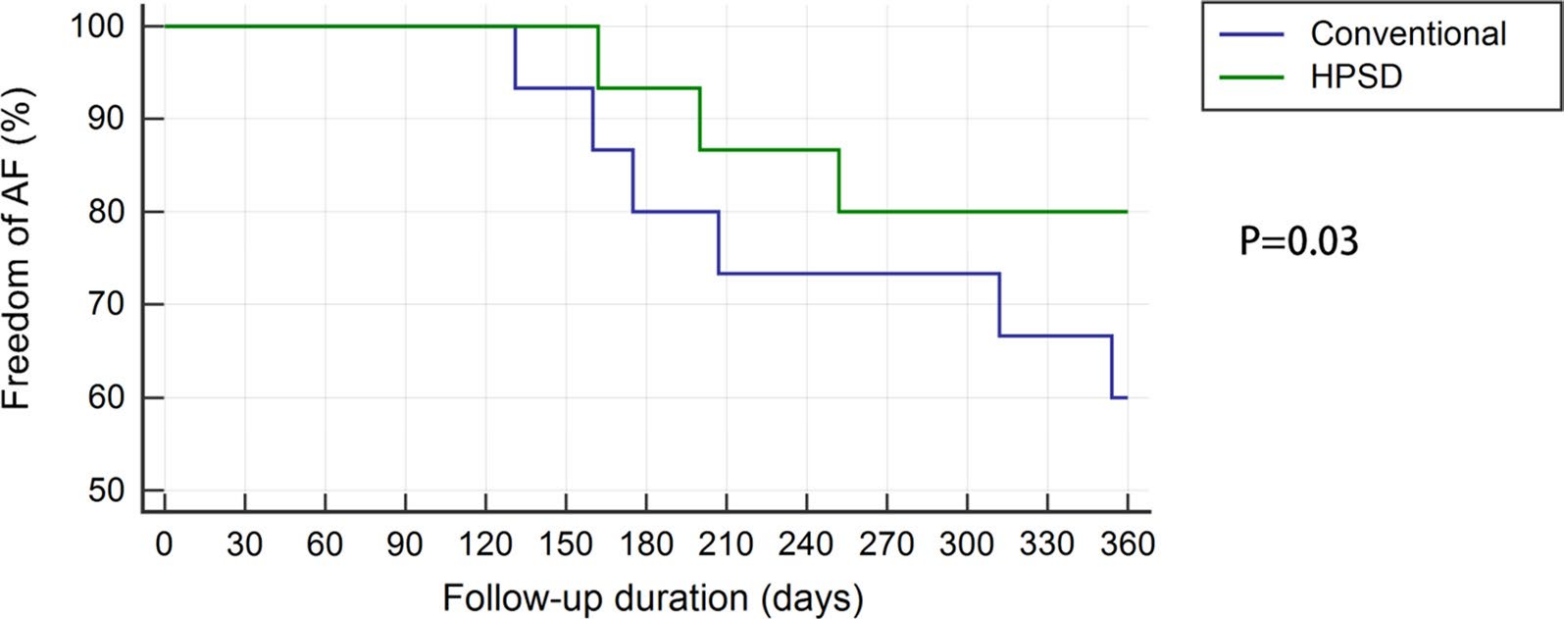
Freedom of AF in all patients

Procedure time and ablation time were significantly shorter in the HPSD group than in the conventional group (91.0 ± 12.1 vs. 124.0 ± 14.2 min, *P* = 0.03; 30.7 ± 19.2 vs. 57.8 ± 21 min, *P* = 0.02, respectively). Two HPSD group patients demonstrated steam pop during ablation and transient elevated contact force; however, the subsequent echocardiography revealed no pericardial effusion. There were 13 patients with recurrent tachyarrhythmia in conventional group (10 were AF and 3 were atypical atrial flutter) and 7 patients with recurrent tachyarrhythmia in HPSD group (5 were AF and 2 were atypical atrial flutter) in a mean 11 ± 1.4 months of follow-up (*P* = 0.03) (Fig. 4).

## Discussion

### Main findings

The main findings of this study included the following: (1) The proportion of optimal lesions created using HPSD ablation was higher than that created using conventional energy setting. (2) The optimal ablation lesions were mostly located at the posterior and anterior walls of the right PV (RPV) in the conventional and HPSD groups, respectively. (3) If lesions achieved the voltage reduction criteria, the effect of FTI on gap formation was insignificant. (4) Gap formation was associated with less voltage reduction during conventional and HPSD ablation. (5) HPSD ablation led to a shorter ablation time and less recurrence rate than did conventional ablation.

### Adequate FTI and durable ablation lesions

Squara et al. found that the cutoff FTI value to best predict transmural lesion creation was ≥ 392 g s and suggested that a high FTI (> 700 g s) warrants transmurality of these lesions (with 100% specificity and positive predictive value) [5]. However, in the current study, the mean FTI for optimal ablation lesion creation that fit the bipolar EGM voltage criteria was found to be only 241 ± 86 g s. In other words, if bipolar EGM voltage changes can be quantified during the ablation procedure with notification of the ablation point already meeting the criteria for optimal lesion creation, the operator may quickly shift to next ablation lesion, rather than maintaining ablation at the same point until the FTI reaches 392 g s or higher. Therefore, this issue may be mitigated using shortening the ablation procedure time.

Of the type B lesions, some lesions represented initial low voltage before ablation. Thus, whether ablation application for these lesions warrants considerations identical to those for type A lesions remains controversial. Nevertheless, here, we provided ablation of 400-g s FTI for the points of these lesions. Moreover, pre- and post-ablation pacing at lesions with initial low voltage may ensure unexcitability; this represents a potential alternative to replace the bipolar EGM transmural criteria [15].

In the present study, we recognized no absolute cutoff values for energy setting at each point when performing ablation for PVI. The real-time observation of the bipolar EGM voltage change during ablation may be a surrogate indicator of optimal lesion creation. Additional studies are required to validate this hypothesis.

### Optimal ablation lesion distribution

Here, a diverse distribution of transmural lesion was noted in both the groups. Most optimal ablation lesions (Type A lesions) were located at the posterior and anterior walls of RPV after conventional energy setting ablation and HPSD ablation, respectively. In addition, the distribution of lesion which did not meet optimal criteria differed between the conventional and HPSD groups. Thus, anatomy may affect ablation efficacy. Chikata et al. [16] noted relatively thin left atrial posterior wall along with relatively low FTI in the left atrial posterior wall. Individualized indicators of optimal lesion creation for different areas of the heart may thus be warranted.

### Safety, ablation time and long-term outcome

In the present study, there were 2 patients occurred steam pop during HPSD ablation and transient elevated contact force. Chen et al. [17] reported 4 cases with steam pops in the first 50 patients because of high catheter contact force. After optimizing the contact force, there was no steam pops occurred in the following procedures [17]. One meta-analysis study concluded that the periprocedural risk is similar between conventional energy and HPSD in AF ablation [18]. Therefore, HPSD is still an effective and safe ablation strategy without increasing the periprocedural risk.

Studies have confirmed the efficacy of short- and long-term outcomes of HPSD ablation [8–10]. Vassallo et al. [19] noted significantly lower procedure time and ablation time after HPSD ablation that that after CF ablation; the authors also noted that the 6- and 12-month recurrence rates were similar in the CF and HPSD groups [19]. In previous study, the short-term outcome was better in the HPSD group than in the conventional group, which could be explained by the higher proportion of transmural lesion created and less gap formation after PVI in the HPSD group [20]. We also noted more detailed differences in lesion characteristics and distribution, potentially providing additional information on differences in the long-term outcome between the 2 ablation strategies. A recent study reported a nonsignificant difference in long-term freedom from AF after HPSD ablation compared with low-power longer-duration ablation [21]. Moreover, HPSD ablation increased the risk of repeat procedure and atypical atrial flutter, possibly because of the relatively low total energy delivered at the left atrial posterior wall [21]. In the current study, most of the lesions at the posterior wall created using HPSD ablation were type B lesions, also indicating relatively low energy delivered at the left atrial posterior wall. Therefore, future studies must focus on optimal settings for HPSD ablation at the left atrial posterior wall.

## Limitation

There are several limitations in the present study. First, this is a retrospective study with relative small numbers of patients, future prospective study in larger study group is warranted to validate the conclusion of present study. Second, methods that improve the stability of ablation catheter such as performing the ablation under general anesthesia and using steerable long sheath were not assessed in the present study. Although, in this study we used non-steerable long sheath during ablation in both conventional and HPSD group. The stability of ablation catheter would influence the lesion creation and might influence the outcome of ablation. Third, the present study aimed to evaluate the lesion characteristic under conventional and HPSD ablation, therefore the lesion index (LSI) was not evaluated in both groups. LSI guided AF ablation especially with high power setting, would be effective and safe [10]. Fourth, the inter-lesion distance is an important factor that might influence the risk of gap formation after ablation. In the present study, there was a consistent inter-lesion distance ≤ 5 mm on 3D Ensite map and this was the same for both groups. Although we did not further analysis the effect of inter-lesion distance, the inter-lesion distance ≤ 5 mm had been supported by the previous report [22, 23]. Fifth, the study numbers are still relatively small to determine the longer term difference in AF outcomes with the two ablation stratagems by using a definition of AF recurrence as > 30 secs but only performing Holter monitors/cardiac event recordings. The possibility of non-detected asymptomatic arrhythmic event might occur during the follow-up period. By using 14-day continuous electrocardiogram patch monitoring or loop recorder, asymptomatic arrhythmia could be more effective detected [24].

## Conclusion

The characteristics of ablation lesions were different between conventional and HPSD energy settings using CF ablation for PVI. Gap formation was related with low voltage reduction during PVI. HPSD ablation led to a shorter ablation time and less recurrence rate than did conventional ablation.

## Data Availability

The datasets used and/or analysed during the current study are available from the corresponding author on reasonable request.

## Abbreviations

AF: Atrial fibrilaltion
HPSD: High-power short-duration
CF: Contact force
PV: Pulmonary vein
PVI: Pulmonary vein isolation
PVP: Pulmonary vein potentials
FTI: Force time integral
EGM: Electrogram
